# Some Physiological and Biochemical Mechanisms during Seed-to-Seedling Transition in Tomato as Influenced by Garlic Allelochemicals

**DOI:** 10.3390/antiox9030235

**Published:** 2020-03-12

**Authors:** Sikandar Hayat, Husain Ahmad, Mubasher Nasir, Muhammad Numan Khan, Muhammad Ali, Kashif Hayat, Muhammad Ali Khan, Farmanullah Khan, Yongqing Ma, Zhihui Cheng

**Affiliations:** 1 College of Horticulture, Northwest A&F University, Yangling 712100, China; 2 College of Natural Resources and Environment, Northwest A&F University, Yangling 712100, China; mubasher@nwafu.edu.cn (M.N.);; 3 Key Laboratory of Urban Agriculture (South), School of Agriculture and Biology, Ministry of Agriculture & Bor S. Luh Food Safety Research Center, SJTU, Shanghai 200240, China; khayat97@hotmail.com; 4 Department of Agriculture, Abdul Wali Khan University, Mardan 23200, Pakistan; malikhan@awkum.edu.pk; 5 Department of Soil and Environmental Sciences, The University of Agriculture, Peshawar 25120, Pakistan; farmankhan@aup.edu.pk; 6 State Key Laboratory of Soil Erosion and Dryland Farming on the Loess Plateau, Institute of Soil and Water Conservation, Northwest A&F University, Yangling 712100, China

**Keywords:** garlic, H_2_O_2_, IAA2, RBOH1, SOD, allelopathy, auxin, biostimulants, defense priming

## Abstract

The effects of aqueous garlic extracts (AGEs), diallyl disulfide (DADS), and allicin (AAS) were investigated during seed-to-seedling transition of tomato. Independent bioassays were performed including seed priming with AGE (0, 100, and 200 µg∙mL^−1^), germination under the allelochemical influence of AGE, DADS, and AAS, and germination under volatile application of AGE. Noticeable differences in germination indices and seedling growth (particularly root growth and fresh weights) were observed in a dose-dependent manner. When germinated under 50 mM NaCl, seeds primed with AGE exhibited induced defense via antioxidant enzyme activities (superoxide dismutase (SOD), peroxidase (POD), and catalase (CAT)), lipid peroxidation (malondialdehyde content (MDA)), and H_2_O_2_ scavenging. Enzyme-linked immunosorbent analysis (ELISA) of the endogenous phytohormones auxin (IAA), abscisic acid (ABA), cytokinin (ZR), and gibberellic acid (GA_3_) in the roots and shoots of the obtained seedlings and the relative expression levels of auxin-responsive protein (IAA2), like-auxin (LAX5), mitogen-activated protein kinase (MAPK7 and MPK2), respiratory burst oxidase homolog (RBOH1), CHI3 and SODCC1 suggested allelopathic functions in stimulating growth responses. Our findings suggest that garlic allelochemicals act as plant biostimulants to enhance auxin biosynthesis and transportation, resulting in root growth promotion. Additionally, the relative expressions of defense-related genes, antioxidant enzymes activities and phytohormonal regulations indicate activation of the defense responses in tomato seedlings resulting in better growth and development. These results, thus, provide a basis to understand the biological functions of garlic allelochemicals from the induced resistance perspective in plants.

## 1. Introduction

To cope with the constantly changing environment, agricultural practices are revolutionized continuously, introducing genetically modified crops, fertilizers, pesticides, fungicides, plant growth regulators, and so on. Their benefits, however, do come at a cost, wherein some are laborious and expensive to practice, while others are considered hazardous from consumer health and environmental safety perspectives on a long-term basis [1,2,3,4]. Thus, the scientific community needs to identify solutions which maximize agricultural production and have limited drawbacks. In this regard, plant growth regulators and biostimulants are gradually becoming the prime research areas among various scientific researchers to enhance plant growth and development [5,6,7]. The growing interest in this area, therefore, demands research that includes both animals and plants. As the word biostimulant implicates, its function primarily involves the stimulation of plant growth and development [8,9] and/or the response to the immediate environment such as stress conditions [10,11] or phytopathogenic invasion [12]. A biostimulant may foster plant growth and development throughout the plant lifecycle from seed germination to plant maturity in a variety of ways, such as enhancing metabolism to improve yield and crop quality, interacting with nutrient assimilation and translocation, facilitating plant defense against adverse conditions, and improving quality attributes such as metabolite abundance, sugar content, fruit color, etc. [5]. To understand the biological function of a biostimulant, it is important to consider the growth stage and pattern, as well as the developmental responses of the subject plants [13]. Seed germination may, therefore, be understood as a vital stage as it reflects the lateral growth pattern of the plants. At this stage, the dormancy status of the seed and the potential for germination are highly regulated by internal and external cues; for example, environmental constrains such as salinity may severely affect the germination process. Similarly, internal cues such as phytohormones and reactive oxygen species (ROS) regulatory networks have vital roles in seed germination and early seedling growth [14,15]. Plant hormones such as abscisic acid (ABA) and gibberellin (GA) are closely associated with seed dormancy and plant responses to environmental stresses, and their relative abundance is, therefore, necessary to indicate germination behavior or responses to stress conditions [16]. Similarly, auxin (IAA) primarily functions in root growth and development. Additionally, IAA regulates expansion and cellular division in the root tips. ROS such as H_2_O_2_ are reported to regulate seed germination by mediating the interaction between ABA and GA. H_2_O_2_ is also considered to accumulate more in germinating seeds in saline conditions than in non-saline conditions. Moreover, ABA and H_2_O_2_ are associated with coordinating the plant defense system by activating mitogen-activated protein kinases MAPKs in plants, leading to an enhanced antioxidant defense system as observed in the leaves of maize [17]. Understanding the transition from seed-to-seedlings also requires investigating the molecular pathways underlying these physiological mechanisms. For instance, the respiratory burst oxidase homologs (RBOHs) are often associated with the production and regulation of ROS, and they can be an essential prerequisite for understanding ROS signaling [18]. Thus, the biological functions of plant biostimulants can be easily speculated from the physiological, biochemical, and molecular responses of the subject plants at a particular growth stage. Depending on the nature, origin, and chemical structure, stimulatory functions of a biostimulant may be established by careful observations of the developmental responses. As biostimulants have the ability to foster plant growth or prime their defense responses to alleviate certain hurdles, their use in vegetable crops seems to be of particular benefit due to the short life-span of the crops. Seed priming or chemical priming, thus, seems to advance the possibility of successful germination and early seedling growth under stress situations.

Previously, numerous biostimulants were introduced from both animal and plant origins [8,9,19,20,21]. Strictly speaking, plant-derived biochemicals are used since ancient civilizations for their therapeutic influence. Garlic is one among these remedial plant species recognized for its beneficial effects throughout the world. It is considered as a broad-spectrum antimicrobial effective against a variety of bacteria, fungi, and viruses [13,22,23]. In addition to its antimicrobial effectiveness, garlic extracts and biochemicals are reported to be involved in a cure for cancer, as well as aid against cardiovascular disorders and blood pressure stabilization [24]. Being a crop, garlic has a repute of strong allelopathic influence on the cropping environment, and garlic allelochemicals are reported to alleviate continuous cropping obstacles in eggplant and cucumber [25,26]. Our previous work on aqueous garlic extracts (AGEs) suggested that the biological influence of garlic is not only limited to allelopathy; it also induces stimulatory responses in treated plants to grow faster and perform better in stress conditions [7,27]. The remedial and allelopathic potentials of garlic, therefore, suggest its possible role as a plant biostimulant; however, it is still unclear if it can stimulate the growth and defense responses of receiver plants during seed-to-seedling transition. Nevertheless, very few scientific evidence is available on the use of garlic allelochemicals to enhance seed germination and early seedling growth of plants. The current research was, therefore, designed to investigate aqueous garlic extract (AGE) and two commercially available allelochemicals (diallyl disulfide (DADS) and allicin (AAS)) as biostimulants to enhance seed germination and developmental responses such as phytohormone regulation, antioxidant enzyme modulation, and relative gene expression signaling during seed-to-seedling transition of tomato. Our studies highlight a comprehensive allelopathic influence of garlic, and they are the first to address the function of garlic allelochemicals as biostimulants for defense priming, as well as improving the germination rate and developmental responses of tomato. The results may, therefore, be considered important in the field of organic agriculture, helping the scientific community to establish garlic-derived botanicals as alternatives to synthetic chemicals with significant use in specialized horticultural situations such as plastic tunnels and glasshouse facilities.

## 2. Materials and Methods

### 2.1. Garlic Extracts and Allelochemical Preparation

Aqueous garlic extracts were prepared from cultivar G-025 obtained from a garlic germplasm unit, horticultural research station, Northwest A&F University, China. The extracts were prepared according to the method described in our previous article [13] to final concentrations of 100 and 200 µg∙mL^−1^. Diallyl disulfide (DADS) was purchased from Sigma Aldrich (Shanghai), and based on the findings of Cheng et al. (2016) [28], diluted to a final concentration of 0.029 µg∙mL^−1^ (0.2 mM). Allicin (ASB-0001535-005) was purchased from ChromaDex International USA, and it was prepared to a final concentration of 0.0039 µg∙mL^−1^ and 100 µg∙mL^−1^ for the current work. Seeds of tomato cultivar Dongyafen Guan were purchased from Yangling, China. 

### 2.2. Experimental Conditions

#### 2.2.1. Bioassays on Tomato Seed Priming with Aqueous Garlic Extracts (AGEs)

Initially, tomato seeds were primed with AGEs for various durations in order to test the priming effects on seed germination indices and post-germination seedling growth characteristics. For priming, tomato seeds were surface-sterilized and dried on a filter paper before being exposed to the aqueous garlic extracts. Priming was carried out using AGEs (0, 100, and 200 µg∙mL^−1^). The 0 µg∙mL^−1^ concentration was considered as a control treatment (CK). Approximately 100 seeds were primed in 20 mL of each concentration for various durations (1, 6, and 12 h). These seeds were further air-dried using a laminar flow unit, and then germination bioassays were performed. To test the priming-induced defense responses, seeds primed with AGEs were subjected to germination under saline conditions (0 and 50 mM NaCl). For seed germination, transparent plastic boxes were used (15 × 15 × 20 cm). Each box contained two layers of filter paper soaked with 5 mL of the stated saline solutions (0 and 50 mM NaCl). Each box contained 50 seeds, and all treatments were replicated four times. These boxes were maintained in a growth chamber (RXZ 500D, Ningbo Jiangnan Instrument Factory, China), in conditions of 26/18 °C (day/night), 70% relative humidity, and white light with an illumination of 30,000 Lux during the day. On alternative days, 2–3 mL of the solutions were added to maintain moisture for the germinating seeds. Seed germination indices, as well as post-germination physiological analysis including antioxidant enzyme assays, malondialdehyde (MDA) content, and H_2_O_2_ accumulation, were investigated to indicate the biostimulation of primed seeds against salt stress. 

#### 2.2.2. Bioassays on Comparative Effects of AGE, Diallyl Disulfide (DADS), and Allicin (AAS) on the Germination and Seedling Growth of Tomato

From the above-mentioned experiments, AGE (100 µg∙mL^−1^) was chosen, and its bioactivity was compared to the commercially available allelochemicals of garlic, diallyl disulfide (DADS (0.029 µg∙mL^−1^)) and Allicin (AAS (0.0039 and 100 µg∙mL^−1^)) in germination bioassays. Tomato seeds were primed for 6 h in these allelochemicals in the stated concentrations and air-dried for 12 h in a laminar flow hood. To indicate the allelochemical effects of AGE, DADS, and AAS on the seed germination and physiological mechanisms of tomato during early seedling growth, another experiment was designed where the seeds of tomato were germinated directly in the presence of these allelochemicals. For this purpose, 50 unprimed tomato seeds were germinated in 5 mL of AGE, DADS, or AAS in the given concentrations. For the control treatment, distilled water was used. On alternative days, 2–3 mL was added to maintain ample moisture conditions.

#### 2.2.3. Germinating Tomato under the Volatile Influence of AGE

To confirm the biological effects of garlic allelochemicals, AGE (100 µg∙mL^−1^) was applied as a volatile medium to the germinating tomato seeds. For this purpose, a double-layered filter paper was pasted onto the lid of the plastic box in which the seeds of tomato were to be germinated. Furthermore, AGE was sprayed on the filter paper until it was soaked just enough such that the extract would not drop down. Then, the tomato seeds were germinated under the volatile influence and, when the filter paper on the lid dried up, it was sprayed with the extract again to maintain the availability of AGE to the germinating seedlings. 

### 2.3. Germination Indices

The germinating seeds were observed on the basis of various indices to indicate the allelochemical influence of garlic allelochemicals. Seeds were considered germinating when the radicle emerged (3 mm). Seed germination indices such as germination percentage (%), germination rate (%), and germination index were recorded for seven consecutive days. 

### 2.4. Morphological Indices during Early Seedling Growth

The fresh weight of germinating seedlings was recorded by weighing 10 seedlings from each replication, and the mean data were subjected to statistical analysis. The hypocotyl and radicle lengths were measured using Vernier calipers, and data were expressed in mm. The seedlings were placed on a black cloth as a background, and photos were taken using a digital camera (Nikon D53, Nikon Corp. Bangkok, Thailand).

### 2.5. Studying Physiology of Obtained Seedlings

#### 2.5.1. Antioxidant Enzymes, MDA, and Soluble Protein Content

Soluble protein content, antioxidant enzyme activities of superoxide dismutase (SOD), peroxidase (POD), and catalase (CAT), and malondialdehyde content (MDA) were observed in the seedlings obtained from priming with AGE. These analyses were performed using previously described methods [13].

#### 2.5.2. H_2_O_2_ Staining to Indicate Salinity Stress Conditions in Seedlings

To identify the stress level, 3,3′-diaminobenzidine tetrahydrochloride (DAB) staining was used to determine the content of H_2_O_2_ in the treated seedlings. Randomly selected seedlings were placed in glass test tubes and immersed in DAB solution for measuring H_2_O_2_. Staining solution was drained after overnight inoculation. Chlorophyll was removed by treating with ethanol and heating in hot boiling water for 10 min. H_2_O_2_ was then observed as a reddish-brown stain after transferring to a paper towel with 60% glycerol [29]. Photographs were taken using a Nikon digital camera, and the abundance of H_2_O_2_ was determined digitally by analyzing the pictures using ImageJ following a published procedure [30].

#### 2.5.3. Enzyme-Linked Immunosorbent Analysis (ELISA) for Hormonal Detection

Plant hormone levels of auxin (IAA), abscisic acid (ABA), cytokinin (ZR), and gibberellic acid (GA_3_) were determined using an enzyme-linked immunosorbent assay (ELISA) according to the methods described by Ren et al. (2018) [31]. To extract plant hormones, 0.5 g of each sample was weighed, quick-frozen, and pulverized. Afterward, 5 mL of 80% methyl alcohol extract solution containing 1 mmol∙L^−1^ butylated hydroxytoluene was added, and the sample was incubated at 4 °C for four hours and then centrifuged at 3500 rpm for 10 min. Then, supernatant was passed through a C-18 solid-phase extraction column and washed successively with 5 mL of 100% methyl alcohol, 5 mL of 100% diethyl ether, and 5 mL of 100% methyl alcohol. Finally, a vacuum desiccator was used to dry the samples and unify the volumes among samples with phosphate buffer (pH 7.5) containing 0.1% Tween-20 and gelatin. The standard solution (IAA, ABA, ZR, and GA_3_) and sample solutions were determined using microtitration plates. Then, 50 μL of standard solution/sample solution and 50 μL of mixed antibodies with a diluted sample were added to ELISA plates, according to the kit label, at 37 °C for 30 min. These plates were washed, and 100 μL of enzyme secondary antibodies were added using the same method, according to the kit label, at 37 °C for 30 min, and the plates were washed again. Then, 100 μL of *o*-phenylenediamine (concentration 1–2 mg∙mL^−1^, with 0.4 μL∙mL^−1^ 30% H_2_O_2_ added after dissolution) was added to each hole, before putting the plate into a wet box when color appeared, using 2 mol∙L^−1^ H_2_SO_4_ to terminate the reaction. Finally, the absorbance was recorded at 490 nm. The analysis of data assumed a logit curve. Each treatment had three biological repetitions and three technical repetitions.

#### 2.5.4. Relative Expression of Genes

The biological effects of AGE and allelochemicals DADS and AAS, as well as the relative expression of numerous genes associated with plant defense and phytohormonal regulation, were observed after seed germination. The observed genes included CHI3 chitinase, auxin-responsive protein (IAA2), like-auxin (LAX5), respiratory burst oxidase homolog (RBOH1), mitogen-activated protein kinase MAPK7 and MPK2 and SODCC1. Primers were designed (Table S1, Supplementary Materials) using Primer3 and Basic Local Alignment Search Tool (BLAST), and the specificity of each primer for its corresponding gene was verified. Methods described by Cheng et al. (2016) [28] were used to extract plant complementary DNA (cDNA) for PCR. Briefly, the expression profiles of selected genes were evaluated in tomato seedlings directly grown under AGE, DADS, and AAS. The total RNA of tomato plantlets was isolated using the Column Plant RNAout (Tiandz Inc., Beijing, China) according to the manufacturer’s instructions and then treated with RNase-free DNase I (Promega, Madison, WI, USA) to remove the contaminating genomic DNA. Reverse transcription (RT) was carried out using the RevertAid RT Reverse Transcription kit (Thermo Fisher Scientific) according to the manufacturer’s instructions. The cDNA was used for real-time fluorescent quantitative PCR (qPCR) with specific primers. The qPCR used was EvaGreen 2× qPCR Master Mix (abm, made in Canada), according to the manufacturer’s guidelines, and the instrument used was an Applied Biosystems Step One Real-Time PCR System (iQ5, Bio-Rad, USA). Data analysis was carried out using the 2^−ΔΔCt^ method and the levels of gene expression relative to the water control [31]. For each gene analysis, three biological replicates were performed.

### 2.6. Data Analysis and Figure Construction

The obtained data were statistically analyzed using Microsoft Excel and Statistix software V.8.1. Data were subjected to analysis of variance (ANOVA), and the differences among the means were analyzed by Tukey’s honestly significant difference (HSD) with *p* ≤ 0.05. The means and standard errors were computed, and figures were constructed using SigmaPlot version 10.0 and Microsoft Excel 2016.

## 3. Results

### 3.1. Seed Priming with Aqueous Garlic Extract (AGE) Improves Seed Germination and Induces Post-Germination Defense System in Tomato

#### 3.1.1. Germination Indices and Morphological Growth of Tomato after Seed Priming with AGE

**Figure 1:**
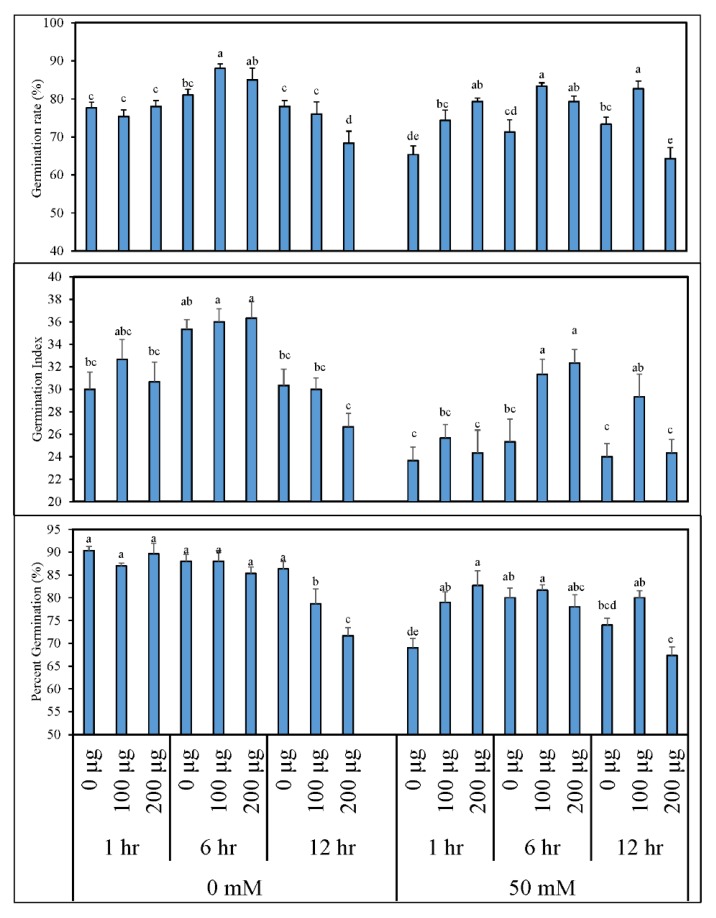
Percentage germination (%), germination rate (%), and germination index of tomato after seed priming with different concentrations of aqueous garlic extract (AGE). Tomato seeds were primed with 0, 100, and 200 µg∙mL^−1^ AGE for 1, 6, and 12 h and germinated under 0 and 50 mM NaCl stress. Obtained data were subjected to analysis of variance (ANOVA) using factorial design. Differences among the means were calculated with Tukey’s honestly significant difference (HSD) (*p* = 0.05). Means followed by the same letters bear no significant difference.

Figure 1 depicts the data for germination percentage (%), germination index, and germination rate (%) of tomato seeds primed with various concentrations of AGE and germinated in normal and saline conditions (0 and 50 mM NaCl, respectively). When tomato seeds were germinated under normal conditions, the germination percentage of the treated plants was no different from the control except when priming for 12 h in 200 µg∙mL^−1^ AGE, which significantly reduced the germination percentage. However, increased germination rate and germination index were recorded when tomato seeds were primed for 6 h in 100 µg∙mL^−1^ AGE. On the other hand, seed priming for 12 h in 200 µg∙mL^−1^ AGE considerably reduced these parameters when compared to other treatments. Effects of seed priming with AGE were, however, more obvious when these seeds were germinated in saline conditions (50 mM NaCl). Due to saline stress, seeds primed with either water or 200 µg mL^−1^ AGE for 12 h exhibited significantly lowered germination percentage, germination rate, and germination index in comparison to the other AGE treatments. Maximum data were recorded for the seeds primed with 100 µg∙mL^−1^ AGE for 6 h with a statistical difference compared to the other treatments in terms of germination rate.

**Figure 2:**
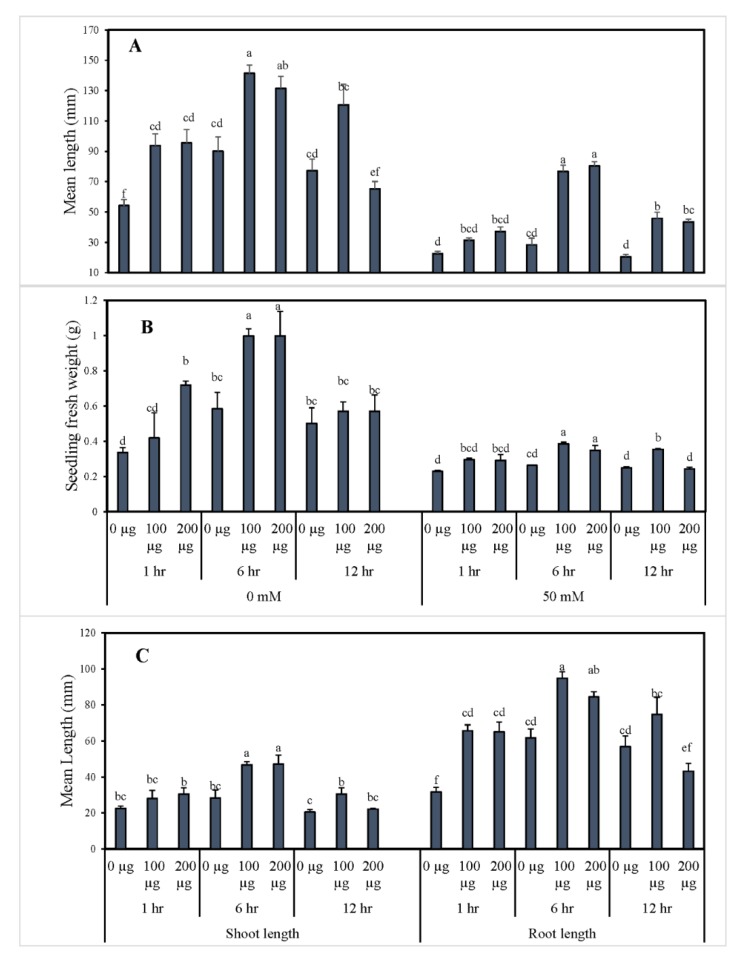
Morphological indices of tomato seedlings obtained from seed priming with AGE. Tomato seeds were primed with 0, 100, and 200 µg∙mL^−1^ AGE for 1, 6, and 12 h and germinated in 0 and 50 mM NaCl stress conditions. Obtained data were subjected to analysis of variance (ANOVA) using factorial design. Differences among the means were calculated with Tukey’s HSD (*p* = 0.05). Bars represent mean values and standard errors for four replicates (10 seedlings per replicate). Means followed by the same letters bear no significant difference.

Similarly, the morphological data for tomato seedlings can be observed in Figure 2. As shown, the seedling’s fresh weight was significantly increased due to seed priming with 100 and 200 µg∙mL^−1^ AGE for 6 h in both 0 and 50 mM NaCl conditions. In contrast, the water control and seeds primed with 200 µg∙mL^−1^ AGE for 12 h both exhibited significantly lower fresh weights whether in 0 or 50 mM NaCl stress conditions. Seed priming produced a staggering improvement in the root length in comparison to the water control. Additionally, the priming duration of 6 h had the maximum root lengths compared to the other durations in both AGE and CK treatments. Furthermore, 100 µg∙mL^−1^ AGE priming for 6 h gave the maximum root length with a statistical difference compared to the other treatments. Similarly, the hypocotyl lengths improved to the maximum (Figure S1, Supplementary Materials) when the seeds were primed with 200 µg∙mL^−1^ AGE for 6 h followed by 100 µg∙mL^−1^ AGE, and the observed improvement showed a statistical difference compared to control treatments. Meanwhile, seed germination under salt stress resulted in reduced seedling growth, and the effects were visible in terms of seedling length. Minimum values were recorded for the seedlings primed with distilled water for 12 h, whereas, the highest values were recorded for the 6-h priming with AGE (100 and 200 µg∙mL^−1^).

#### 3.1.2. AGE Priming Alleviated Salt Stress during Germination by Affecting Reactive Oxygen Species (H_2_O_2_) Levels in Tomato Seedlings

**Figure 3:**
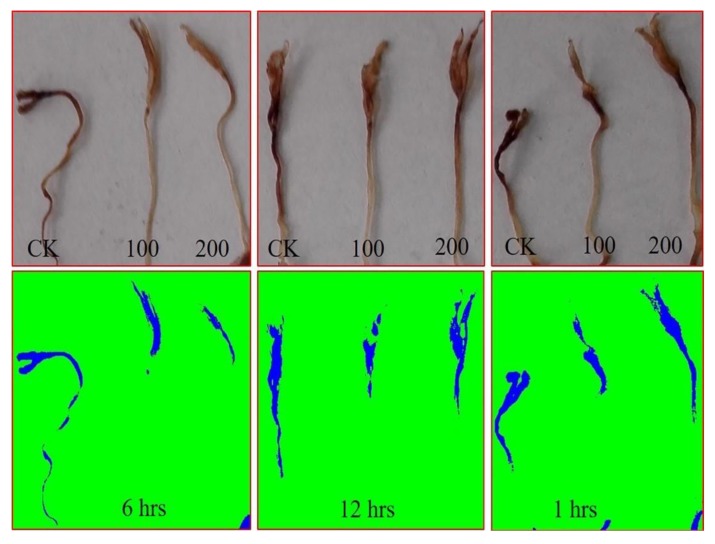
H_2_O_2_ abundance in tomato under 50 mM NaCl. Tomato seeds were primed with 0, 100, and 200 µg∙mL^−1^ AGE for 1, 6, and 12 h and germinated under 50 mM NaCl stress. The abundance of H_2_O_2_ was visualized by 3,3′-diaminobenzidine tetrahydrochloride (DAB) staining. Images were analyzed using ImageJ software.

The H_2_O_2_ accumulation in the seedlings germinated under salt stress is depicted in Figure 3.

Image analysis revealed considerably higher abundance of H_2_O_2_ in the control (water-treated) seedlings, indicating oxidative stress. However, seed priming with AGE showed low levels of H_2_O_2_ accumulation, which clearly reflects that AGE seed priming successfully attenuated the stress levels in the resulting seedlings. Priming tomato seeds for 6 h considerably reduced H_2_O_2_ accumulation as compared to the other priming durations, which corroborates the other observations in the experiment. Nevertheless, seed priming with 200 µg∙mL^−1^ AGE for 12 h resulted in a relatively higher level of H_2_O_2_ accumulation, which indicates the detrimental effects of AGE at higher concentrations.

#### 3.1.3. Seed Priming Improves Physiological Responses of Tomato Seedlings

**Figure 4:**
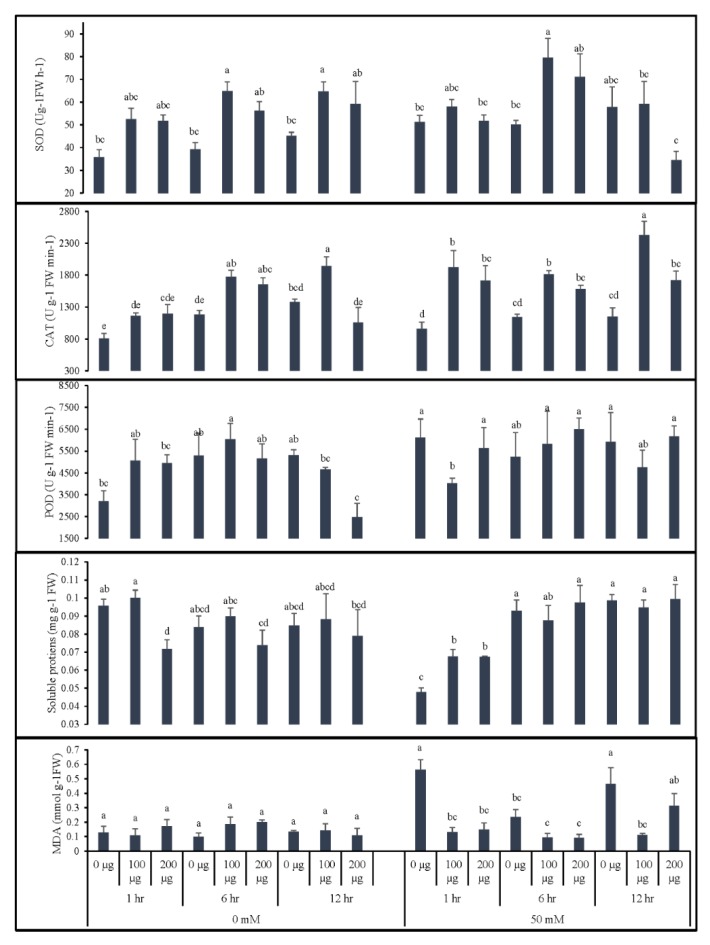
Effect of AGE priming on the antioxidant enzyme activities, lipid peroxidation, and soluble protein abundance of tomato. Primed seeds were germinated under 0 and 50 mM NaCl stress. Data for antioxidant enzyme activities of catalase (CAT), peroxidase (POD), and superoxide dismutase (SOD), malondialdehyde content (MDA), and soluble protein abundance are presented as means + standard errors (bars). All data were subjected to factorial ANOVA and post hoc Tukey’s honestly significance difference (HSD) at *p* = 0.05. Means followed by the same letters bear no statistical difference.

As depicted in Figure 4, both priming duration and AGE concentration affected antioxidant enzyme activities (superoxide dismutase (SOD), peroxidase (POD), catalase (CAT)), lipid peroxidation (MDA content), and the soluble protein abundance of tomato seedlings; moreover, the effects varied when seeds were germinated in normal and saline conditions.

When the seeds were germinated in normal conditions (0 mM NaCl), a slight increase in POD activity was observed; however, the highest concentration of AGE (200 µg∙mL^−1^) significantly reduced POD activity with prolonged priming of 12 h. Similar effects were observed for soluble proteins, where seeds underwent prolonged exposure to a higher concentration of AGE. On the other hand, seed priming with 100 µg∙mL^−1^ AGE for 6 h increased CAT activity in comparison to control treatments, whereas prolonged priming (12 h) in the highest concentration of AGE (200 µg∙mL^−1^) significantly reduced the activity of this enzyme. Seed priming with AGE also had increasing effects on the SOD activity with a statistical difference compared to control treatments. Seed priming with AGE resulted in a slightly increased but non-significant influence on MDA content in comparison to the control seedlings.

Data obtained from seedlings germinated in 50 mM NaCl revealed that seed priming with AGE can effectively induce the antioxidative responses of tomato plants. Significantly higher levels of SOD and CAT were observed in the seedlings obtained from priming with 100 µg∙mL^−1^ for 6 h and 12 h, respectively. We observed no significant differences among the treatments for POD activity except for 1-h seed priming with 100 µg∙mL^−1^ AGE, which resulted in lowered POD activity. Interestingly, the abundance of the soluble protein was significantly reduced when seeds were primed for 1 h, with the control having the lowest data. The other durations, however, had no significant differences. Seed germination in 50 mM salt resulted in high levels of MDA content in the control seedlings. Seed priming with AGE, however, significantly influenced the MDA content with statistically low levels in comparison to the control seedlings.

### 3.2. Allelopathic Effects of AGE, Diallyl Disulfide, and Allicin on Tomato Seed Germination and Post-Germination Phytohormonal Regulation

In this experiment, 100 µg∙mL^−1^ AGE was further compared with allicin (AAS) and diallyl disulfide (DADS) to understand the difference in allelopathic functions of garlic-based compounds on seed germination and seedling development of tomato in various bioassays, and the results are presented below.

#### 3.2.1. Germination Indices and Post-Germination Morphological Observations

**Figure 5:**
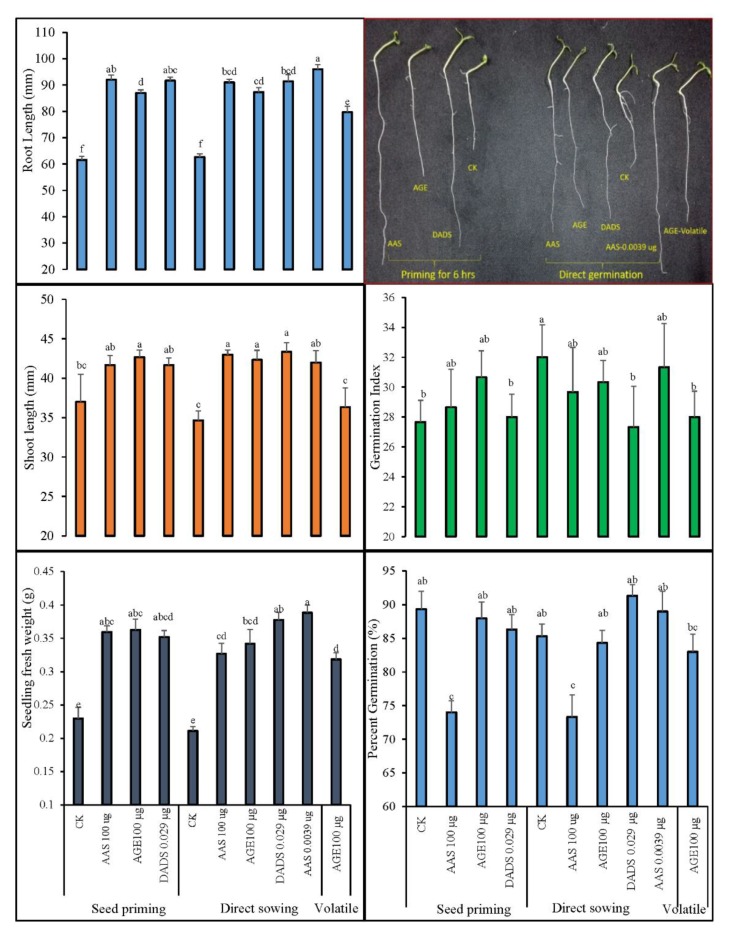
Germination indices and post-germination morphology of tomato after different treatments of AGE, diallyl disulfide (DADS), and allicin (AAS). Germination percentage, germination index, seedling length (cm), and fresh weight (g) are depicted. Tomato seeds were primed with 100 µg∙mL^−1^ AGE, 100 µg∙mL^−1^ allicin (AAS), or 0.029 µg∙mL^−1^ DADS for 6 h. Distilled water was taken as the control treatment. In another set, tomato seeds were sown directly under the allelochemical effects of these chemicals including 0.0039 µg∙mL^−1^ AAS. Additionally, 100 µg∙mL^−1^ AGE was also separately provided as the volatile germinating medium to the seeds. Obtained data were subjected to analysis of variance (ANOVA) using factorial design. Differences among the means were calculated with Tukey’s HSD (*p* = 0.05). Bars represent mean values and standard errors for four replicates. Means followed by the same letters bear no significant difference.

The data for germination percentage (%), germination index, seedling fresh weight (g), and root and shoot length (mm) of tomato seedlings germinated under different treatments of AGE (100 µg∙mL^−1^), DADS (0.029 µg∙mL^−1^), or AAS (100 and 0.0039 µg∙mL^−1^) are provided in Figure 5.

Statistical analysis indicated a significant reduction in the germination percentage and germination index of tomato seeds with the application of a high concentration of AAS (100 µg∙mL^−1^), whether as seed priming or as an active medium, in comparison to the other treatments. However, among the other treatments, only AGE and a lower concentration of AAS (i.e., 0.0039 µg∙mL^−1^) improved the germination index, but the effects were not significantly different. The effects of treatments were, however, quite obvious on the morphology of the obtained tomato seedlings. A significant increase was observed in the root lengths, and maximum data were recorded for the seedlings germinated under 0.0039 µg∙mL^−1^ allicin. Similar effects were noted when the data for shoot lengths were compared among the treatments. Garlic chemicals also improved the fresh weight of tomato seedlings when the seeds were primed, directly germinated, or germinated under the volatile application of AGE. The fresh weights were higher than the control seedlings with a statistical difference.

#### 3.2.2. Influence of Various Treatments on the Endogenous Hormonal Levels

**Figure 6:**
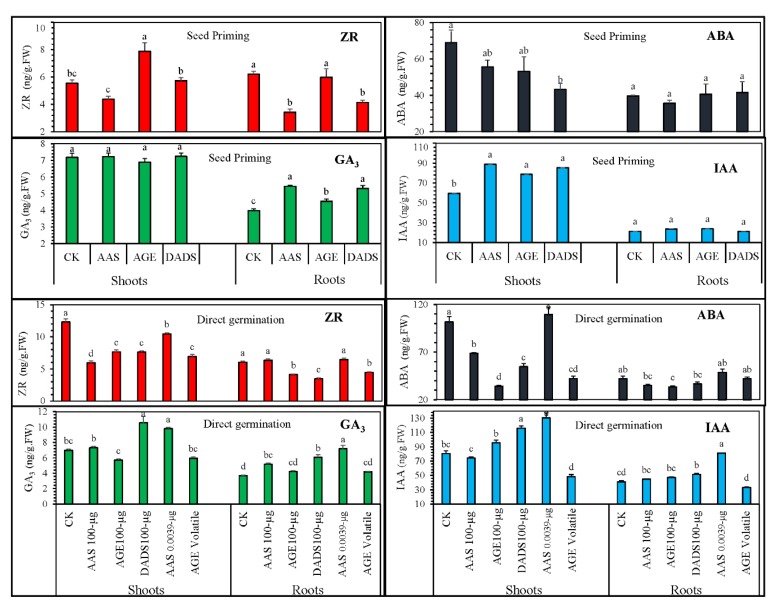
Post-germination phytohormonal regulation in tomato shoots and roots. The endogenous phytohormones auxin (IAA), gibberellic acid (GA_3_), cytokinin (ZR), and abscisic acid (ABA) are presented. Tomato seeds were germinated after seed priming or, under an allelochemical medium of 100 µg∙mL^−1^ AGE, 0.29 µg∙mL^−1^ DADS, or AAS (100 and 0.00039 µg∙mL^−1^). Bars represent means and standard errors after ANOVA and HSD test.

Figure 6 depicts the results for endogenous phytohormones observed in tomato seedlings obtained from seed priming. Statistical analysis showed that garlic allelochemicals significantly regulated these phytohormones. Seed priming with AGE and AAS resulted in significantly different cytokinin (ZR) levels than control or DADS treatments. On one hand, ZR levels in hypocotyl increased with AGE and AAS, whereas DADS reduced the ZR level, suggesting a contrasting effect among these chemicals. ZR levels in the roots reduced significantly in the seeds primed with AAS or DADS, whereas AGE-treated seedlings did not show any significant differences. Likewise, priming with AGE or allelochemicals (AAS and DADS) significantly increased the shoot hormonal levels of IAA compared to control seedlings, whereas no significant difference was observed for IAA levels in the roots. Additionally, priming did not influence the GA_3_ levels in the shoots, while, in roots, AAS, DADS, and AGE treatment produced an increasing effect on the GA_3_ levels with a statistical difference compared to control treatment. The ABA levels in tomato shoots were interestingly mediated after DADS priming. Both AGE- and AAS-treated seedlings also exhibited decreasing a trend for ABA; however, the effect was not significant compared to control seedlings. No obvious difference was observed in the root ABA levels among the treatments. When tomato seeds were germinated under garlic allelochemicals, the endogenous hormones levels were statistically different compared to the control. Levels of ABA were significantly decreased, with AGE having the strongest effect, whereas 0.0039 µg∙mL^−1^ AAS did not produce any significant effect on ABA levels. Similarly, AGE significantly reduced the ABA levels in roots, whereas the other treatments showed non-significant effects. AGE, DADS, and AAS had a reducing effect on the ZR levels, with 100 µg∙mL^−1^ AAS having significantly lowered values. Compared to the control, the ZR level of tomato germinated under volatile AGE was also significantly lower. AAS had a dose-dependent effect, and the ZR levels decreased with increasing concentration of AAS. In the roots, only AGE, DADS, and the AGE volatile medium significantly reduced the ZR content compared to control treatments. Increased IAA levels were observed under AGE, DADS, or AAS (0.0039 µg∙mL^−1^), where AAS (0.0039 µg∙mL^−1^) showed the highest effects, followed by DADS treatment. The lowest value was recorded for the seedlings under volatile AGE. Similar data were observed for the IAA levels of roots, whereas AGE as a volatile medium comparatively reduced the IAA level. Highest values for GA_3_ were recorded for DADS, followed by 0.0039 µg∙mL^−1^ AAS. AGE, on the other hand, reduced GA_3_ levels compared to control treatment. AGE, DADS, or AAS, however, increased the root GA_3_ level compared to the control treatment, and the highest value was observed for 0.0039 µg∙mL^−1^ AAS, followed by DADS treatment.

#### 3.2.3. Relative Expression of Genes under AGE, DADS, and AAS Treatment

**Figure 7:**
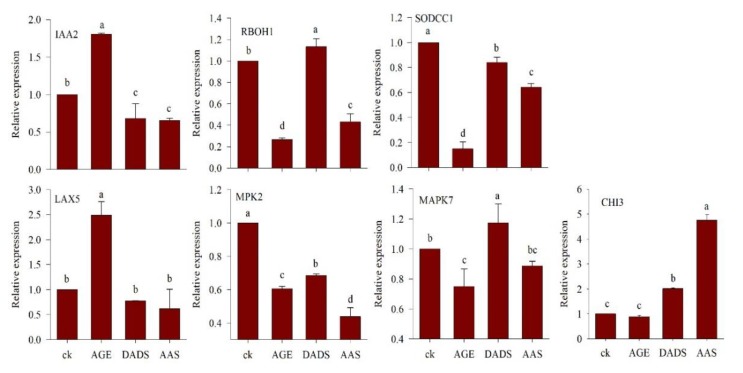
Relative expression of various genes in tomato seedlings germinated after priming with AGE (100 µg∙mL^−1^), DADS (0.029 µg∙mL^−1^), and AAS (100 µg∙mL^−1^) relative to water control (1.0). The relative expressions of different genes are presented as means and standard errors of three biological replications. Data were subjected to ANOVA and the comparison to control was analyzed using the least significant difference (LSD) and Dunnett’s multiple comparison with a control test.

Figure 7 indicates the relative expression levels of various genes of treated plants compared to the control. Statistical analysis revealed that treatment with garlic allelochemicals affected the relative gene expression of RBOH1 when compared to the control. The difference in expressions levels based on the Dunnett’s multiple comparison with a control test were −0.7338*, 0.1336*, and −0.5686* (AGE, DADS and AAS respectively) in comparison to that of control (1.0). The relative expression levels for IAA2 in the treated seedlings were 0.8061*, −0.3208*, and −0.3429* for AGE, DADS and AAS respectively. Only AGE treatment resulted in significantly different relative expression of LAX5 in the tomato seedlings (1.488*). The other two treatments (DADS and AAS) affected the relative expression showing values −0.2274 and −0.38-fold of the control (1.0), and were non-significant. The results for the expression analysis of MAPK7 genes also indicated that, among the treatments, only DADS could significantly alter the relative gene expression to −0.2513* in comparison to the control, whereas the other allelochemicals did not induce any significant results. Statistical analysis of the relative gene expression data for MPK2 indicated a significant effect of garlic allelochemicals. The relative expression in comparison to the control was −0.3951* for AGE, −0.3155* for DADS, and −0.5613* for AAS. No significant difference was observed for the relative expression level of CHI3 in tomato seedlings obtained from AGE treatment whereas, the rest of treatments i.e., DADS and AAS significantly altered the relative expression levels to 1.0127* and 3.754* respectively. There was a significant effect of AGE, DADS and AAS treatment on the expression level of SODCC1. When compared to the control, the relative expression levels were −0.8502*, −0.1603* and −0.3571* respectively.

## 4. Discussion

Current findings suggest garlic extracts and allelochemicals (DADS and AAS) as plant biostimulants to enhance seed germination and post-germination growth of tomato. Seed priming with AGE resulted in a speedy germination, and the effect was closely associated with the concentration of AGE and the duration of seed priming. Seeds primed with AGE for 1 and 6 h showed an overall improved response; however, exposure to a higher concentration for a longer time (12 h) subdued germination indices. These findings agree with the previously reported inhibitory effects of higher concentrations of garlic chemicals on the seed germination and growth of cucumber and tomato [28,31]. The biochemical composition of garlic, such as starch content, vitamins, saponins, and traces of minerals, seems to have a positive influence on the emerging tomato seedlings. However, strictly speaking, freshly prepared garlic extracts contain organosulfur compounds, primarily “allicin” [13], which are highly bioactive and permeable through biological membranes [32]. The active permeability, thus, provides a plausible explanation for the capricious germination indices in relation to the variable concentrations of AGE, i.e., when the threshold levels are exceeded, the organosulfur compounds may inhibit the normal germination process inside tomato seeds. Apart from the dose-dependent germination improvement, we noticed that tomato seeds primed for 6 h had generally enhanced germination indices in comparison to the other durations, which indicates that tomato seed priming for 6 h can effectively enhance the germination and early seedling growth. However, this speculation requires a comparable investigation on different varieties of tomato. In addition to accelerated germination, AGE priming also improved growth of the obtained tomato seedlings, particularly the root growth and fresh weights, supporting earlier studies on the stimulatory effects of AGE on various vegetables such as tomato, pepper, eggplant, and cucumber [7,13,19,27]. When germinated in 50 mM NaCl, the AGE-primed tomato seeds significantly survived compared to the inhibited growth of control seedlings. The effects were visualized based on the abundance of H_2_O_2_ accumulated in the control and treated seedlings. H_2_O_2_, as a representative of ROS, is generally considered to accumulate higher in germinating seeds under saline conditions than in non-saline conditions, and it is often corelated with delayed germination [33]. In addition, ROS are often considered important signaling molecules to stimulate developmental responses in plants. Moreover, they can act as indicators of stress severity in plants under stress conditions, however, their overproduction may interact with normal cellular processes and can destroy cellular compartments or denature DNA [34]. The use of antioxidant enzymes such as superoxide dismutase (SOD), peroxidase (POD), catalase (CAT), ascorbic peroxidase (APX), etc. is, thus, a natural control strategy of plants to limit the damage due to ROS [29,35]. Biochemical analysis revealed a higher abundance of antioxidant enzymes in the treated seedlings with lower levels of malondialdehyde (MDA) content. This finding suggests that seed priming with AGE induced resistance responses of the emerging tomato seedlings, thereby limiting the ROS (H_2_O_2_) damage by preventing lipid peroxidation. Previously, foliar application and root fertigation with AGE resulted in elevated resistance in pepper plants against *Phytophthora capsici* infection with lower levels of H_2_O_2_ abundance [7], which agrees with the finding of induced resistance produced by AGE in the current work. Tomato seeds treated with AGE and germinated under non-saline conditions showed slightly increased antioxidant enzyme activity, indicating a probable increase in ROS such as O_2_^−^ and H_2_O_2_. It can be speculated that abundant ROS actively participated in the germinating seeds’ biochemical processes, while the increased antioxidant enzymes effectively regulated the overproduction of ROS, thereby protecting the seedlings from oxidative stress damage. Nevertheless, no significant increase was noted for SOD abundance when the water-treated (control) seeds were germinated under saline conditions. When correlated to the significantly increased MDA content, a speculation can be drawn that salinity may have subdued the capacity of this antioxidant enzyme due to the oxidative damage, and the resulting seedlings were no longer able to cope with the overwhelming abundance of the ROS.

During seed germination and early seedling growth, complex biochemical processes are involved, including hormonal crosstalk and signaling, protein synthesis, reactive oxygen species regulatory networks, etc., and any disturbance in their balance can essentially influence the growth and development of the resulting seedlings [33,36,37]. The relative abundance of endogenous hormones such as auxin (IAA), gibberellic acid (GA_3_), cytokinin (ZR), and abscisic acid (ABA) in tomato seedlings was altered due to garlic treatment (seed priming, direct germination, or volatile application). High levels of IAA were detected in tomato seedling shoots in comparison to the roots. Furthermore, the abundance was higher in seedlings germinating under the direct influence of these chemicals than under seed priming or volatile application, an indication of the close association of garlic chemicals with auxin biosynthesis. Previously, DADS was shown to allelopathically improve root growth, as well as mitotic cellular division and expansion, through regulation of auxin in cucumber and tomato seedlings [28,31], thus supporting the current results. Auxin is considered central to a myriad of aspects of plant growth and responses, both on a cellular and a whole-plant level [16]. Its role is generally established in cell division, expansion and differentiation [14]. Whereas, on the whole-plant level, it functions in apical dominance, root growth, and characteristics [37]. The promotion of root growth in the treated plants is likely the result of elevated levels of auxin. On the contrary, cytokinin (ZR) levels were reduced due to garlic treatments. This offers a plausible explanation for the promoted root growth, as IAA is involved in root elongation and development, whereas ZR is reportedly involved in inhibition [38]. Furthermore, AGE, DADS, and AAS particularly altered the ABA and GA_3_ levels. Both of these phytohormones act in seed dormancy and plant responses to stress conditions [39,40]. ABA is generally responsible for seed dormancy, whereas GA_3_ functions in dormancy release and onset of germination [16]. The active influence of garlic allelochemicals on these phytohormones clearly suggests the allelopathic functions in stimulating germination processes in the seeds. Apart from its function in seed dormancy, ABA signaling is also associated with crosstalk involving other hormonal pathways and reactive oxygen species, which can attenuate the activity of this central inhibitory factor [40,41,42,43]. As discussed earlier, the AGE-treated tomato seeds indicated elevated H_2_O_2_ levels in non-saline conditions. The role of H_2_O_2_ in mediating the interaction between ABA and GA during alleviation of dormancy and seed germination in *Arabidopsis* [33] provides an explanation to understand the biological functions of garlic chemicals in the developmental responses of obtained tomato seedlings. Similarly, GA_3_ plays a vital role in the developmental responses of plants such as leaf expansion and trichome development [38], which supports the idea of its involvement in the observed pronounced growth of tomato seedlings. Having said that, the bioactivity of garlic allelochemicals cannot be absolutely confined to these physiological mechanisms, as there are other key factors which regulate seed germination such as ethylene and nitric oxide (NO) [34]. This represents a key gap in our understanding of the biological functions of these allelochemicals, and future research may pave the way to fully understanding their modes of action in seed germination.

Current results also indicated the bioactivity of garlic allelochemicals with respect to molecular levels by altering the relative expression levels of various genes. In addition, expressions were different in response to treatments, indicating that the particular biological function of each compound may vary in some cases. Respiratory burst oxidase homologs (RBOHs) often function as signaling biological processes in plants, and they are associated with ROS production [41,42]. The relative expression levels of RBOH1 were altered in the treated seedlings observed in the current study. RBOH-mediated ROS production was previously shown to facilitate lateral root emergence in *Arabidopsis* [43]. In addition to their role in cellular growth and expansion, RBOHs mediate the plant abiotic stress response and adaptations [44], associated with the ABA signaling network and ROS regulation. The differential expression levels of RBOH1 in the treated plants indicate the biological function of garlic chemicals in the crosstalk of ABA and RBOH signaling pathways, thereby mediating ROS production during early seedling growth. In a previous study, silencing RBOH1 and MPK2 was reported to abolish brassinosteroid-induced H_2_O_2_ generation and tolerance of tomato plants to heat stress [44]. The increased resistance of treated plants in the current study is, therefore, likely due to the bioactivity of garlic compounds on a molecular level. Treated plants had altered relative expression levels of auxin-responsive protein (IAA2) and like auxin (LAX5). LAX genes are generally associated with auxin influx [45]. They encode proteins involved in the shoot apical meristem and root primordia, which are important in auxin transport. It is evident from the current results that garlic allelochemicals (organosulfur compounds) function as signaling molecules initiating ROS signaling and auxin biosynthesis, thereby improving root growth and development. Cheng et al. (2016) [28] reported the upregulation of IAA2 and LAX5 in tomato when treated with DADS, which supports the idea of bioactivity of this allelochemical in the genetic responses of tomato. Altered levels of the relative expressions of CHI3, MPK2, SODCC1, and MAPK7 further advanced the biological functions of garlic allelochemicals in the induced defense responses of tomato seedlings. These genes are well established for their functions in plant responses to stress. Previously, inoculation with rhizosphere bacteria induced systemic resistance in tomato plants with elevated levels of CHI3 against fungal infection [46]. Similarly, mitogen-activated protein kinases (MAPKs) and MPKs have a function in resistance against a variety of stress conditions [47,48,49]. Due to the altered relative expression of these defense-related genes, we speculate that, in the current study, treatments of garlic chemicals acted as a stress stimulus which altered the molecular functions, thereby activating the defense mechanism and associated gene signaling in tomato seedlings. Nevertheless, altered ABA, IAA, and GA_3_ levels due to AGE, DADS, or AAS treatments were observed. Hormonal signaling helps plants to respond to stress conditions, which includes regulating molecular pathways [40,50]. MAPKs regulate diverse cellular programs by relaying extracellular signals to intracellular responses, and they are often suggested to function in plant responses to stress conditions [51]. As discussed above, DADS was already reported to function in root development; however, our results demonstrate that DADS, allicin, and AGE act as plant biostimulants to boost seed germination and enhance physiological functions such as phytohormones and molecular signals. Seed germination is understood as the primary process in the lifecycle of plants that determines the proceeding growth and development [52]; thus, it is rational to consider that alterations during seed germination may affect the lateral growth of the plants. Altogether, the current findings suggest the potential of garlic-derived biochemicals to enhance the germination process by inducing numerous physiological mechanisms. By inducing antioxidant enzymes, ABA signaling, and GA_3_, as well as mediating RBOH1 or MPK2, these allelochemicals seem to have enabled tomato seeds to overcome the germination hurdle of salinity, which inhibited the germination of the control seeds. As mentioned earlier, garlic allelochemicals are bioactive and able to penetrate through biological membranes, with the current results providing evidence for their biostimulatory effects, involving the regulation of plant physiological functions. So far, no scientific work showed the biological functions of allicin or AGE in the seed-to-seedling transition of tomato plants from the perspective of induced resistance. The current findings may, therefore, be considered the first to establish the biological functions of these chemicals from a seed priming perspective. The findings of the current work suggest the possibility of garlic-based preparations to improve the growth and developmental of vegetables during early growth stages; however, due to the complex biochemical composition of garlic extracts and the differential relative expressions of the studied genes, further research is required to indicate the exact mode of action of these particular chemicals.

## 5. Conclusions

Based on the current results, it is thoughtfully concluded that garlic extracts (AGEs) and allelochemicals diallyl-disulfide and allicin (DADS and AAS) are bioactive compounds to stimulate physiological and biochemical mechanisms of tomato plants during seed-to-seedling transition. Tomato seed priming with AGE for 6 h can induce defense responses to overcome germination hurdles such as mild salt stress. Furthermore, the biological effects of garlic-based compounds are closely associated with plant antioxidant enzymes (SOD, POD, and CAT) and ROS scavenging, which contribute to the defense mechanism. The allelopathic influence of garlic extracts and allelochemicals resulted in significant phytohormonal regulation of IAA, GA_3_, ZR, and ABA which correlated to root-growth improvement during the early growth stages. Additionally, the relative genetic expression of IAA2, CHI3, SODCC1, MPK2, MAPK7, LAX5, and RBOH1 was significantly different in treated plants, indicating garlic allelochemicals act as signaling molecules in plant functional processes. It is likely that organosulfur compounds of garlic can act as plant biostimulants to improve early seedling growth and development of tomato. Additionally, these results provide a basis for studying the biological functions of garlic allelochemicals (particularly organosulfur compounds) as next-generation biostimulants in agriculture. Nonetheless, these findings are vital for the preparation of garlic-based compounds to enhance vegetable seed germination and early growth, particularly under adverse environmental conditions such as in saline areas.

## Supplementary Materials

The following are available online at https://www.mdpi.com/2076-3921/9/3/235/s1: Figure S1. Effect of AGE priming on the morphological growth of tomato seedlings; Table S1. Primers designed for various genes studied.
